# Full-Length Transcriptome Profile of *Apis cerana* Revealed by Nanopore Sequencing

**DOI:** 10.3390/ijms251910833

**Published:** 2024-10-09

**Authors:** Xiao-Fen Hu, Meng-Jie Jin, Zhi-Xian Gong, Zong-Liang Lin, Li-Zhen Zhang, Zhi-Jiang Zeng, Zi-Long Wang

**Affiliations:** 1 Jiangxi Province Key Laboratory of Honeybee Biology and Beekeeping, Jiangxi Agricultural University, Nanchang 330045, China; hxfen999@aliyun.com (X.-F.H.); mengjiejin@icloud.com (M.-J.J.); ling15350309823@stu.jxau.edu.cn (Z.-X.G.); zlzcg@126.com (L.-Z.Z.); bees@jxau.edu.cn (Z.-J.Z.); 2 Honeybee Research Institute, Jiangxi Agricultural University, Nanchang 330045, China; 3 College of Animal Science and Technology, Jiangxi Agricultural University, Nanchang 330045, China

**Keywords:** *Apis cerana*, full-length transcriptome, alternative splicing, nanopore sequencing, differentially expressed transcripts

## Abstract

The Asian honey bee (*Apis cerana*) plays a crucial role in providing abundant bee products and in maintaining ecological balance. Despite the availability of the genomic sequence of the Asian honey bee, its transcriptomic information remains largely incomplete. To address this issue, here we constructed three pooled RNA samples from the queen, drone, and worker bees of *A. cerana* and performed full-length RNA sequencing using Nanopore single-molecule sequencing technology. Ultimately, we obtained 160,811 full-length transcript sequences from 19,859 genes, with 141,189 being novel transcripts, of which 130,367 were functionally annotated. We detected 520, 324, and 1823 specifically expressed transcripts in the queen, worker, and drone bees, respectively. Furthermore, we identified 38,799 alternative splicing (AS) events from 5710 genes, 44,243 alternative polyadenylation (APA) sites from 1649 gene loci, 88,187 simple sequence repeats (SSRs), and 17,387 long noncoding RNAs (lncRNAs). Leveraging these transcripts as references, we identified 6672, 7795, and 6804 differentially expressed transcripts (DETs) in comparisons of queen ovaries vs drone testes, worker ovaries vs drone testes, and worker ovaries vs queen ovaries, respectively. Our research results provide a comprehensive set of reference transcript datasets for *Apis cerana*, offering important sequence information for further exploration of its gene functions.

## 1. Introduction

*A. cerana* is an important bee species widely cultivated in Asia and plays a crucial role in beekeeping. It exhibits desirable traits such as strong resistance to mites, heightened olfactory sensitivity, and efficient collection of scattered nectar sources [1]. To date, several versions of the Asian honey bee genome and its predicted genes have been published [2,3,4]. Alongside, several studies on the transcriptome of the Asian honey bee have been conducted [5,6]. Even though, obtaining the complete full-length transcriptome sequences for *A. cerana* remains an essential endeavor.

The transcriptome encompasses the entirety of the RNA molecules transcribed from specific tissues or cells during a particular developmental stage or functional state, including mRNA, rRNA, tRNA, lncRNA, etc. [7]. Transcriptional regulation is a pivotal step in gene expression regulation in eukaryotes. Alternative splicing represents a significant post-transcriptional processing mode that greatly augments the diversity of transcriptomes and proteomes at the cellular, tissue, and individual levels [8,9]. It is widespread in eukaryotes; for instance, over 95% of multi-exon genes in humans [10], 60% in *Arabidopsis thaliana* [11], and 30% in pigs [12] undergo alternative splicing. Extensive research substantiates the involvement of alternative splicing in regulating spatiotemporal specificity, growth and development, stress response, and environmental adaptation, as well as disease occurrence [13,14].

Transcriptome sequencing facilitates the comprehensive and rapid acquisition of expression information pertaining to nearly all transcripts within specific organs or tissues of a species under the given conditions. So far, second-generation sequencing technologies are extensively employed in transcriptome studies [15,16]. However, due to their limited read length (100–150 bp), complete transcript sequences can only be obtained by assembling and merging short reads. Unfortunately, transcript assembly frequently results in incompleteness or even inaccuracy, thereby affecting subsequent investigations into gene structures, such as alternative splicing and fusion genes. Additionally, the sequencing of complex regions characterized by highly repetitive or GC-rich sequences has proven to be challenging. The newly developed third-generation sequencing technologies do not require PCR amplification during the sequencing process. These technologies can sequence individual DNA (or RNA) molecules and generate long reads, avoiding potential PCR amplification errors and biases and facilitating the identification of different splicing variants of genes. They also retain the high throughput and cost-effectiveness similar to second-generation sequencing technologies. Currently, there are two commonly used third-generation sequencing technologies, including Single-Molecule Real-Time (SMRT) technology by Pacific Biosciences [17] and nanopore sequencing by Oxford Nanopore Technologies [18].

Nanopore sequencing technology is based on a special synthetic polymer membrane placed in an ionic solution. This membrane is uniformly distributed with modified transmembrane channel proteins known as nanopores, which have diameters that allow the passage of only a single nucleotide polymer [18]. These nanopore proteins act as reader proteins. When a potential difference is applied on both sides of the membrane to generate an electric current, with the help of motor proteins, the DNA strands are unwound and pass through the nanopores in the polymer membrane. Since the four nucleotide bases (A, C, G, and T) possess distinct electrical properties, differences in electrical signals can be exploited to detect specific bases as they traverse the nanopore, thus enabling sequencing. Nanopore sequencing technology has the merits of rapid, real-time analysis and long read lengths. It has broad applications in whole-genome sequencing, comparative genomics, macrogenomics, epigenetics, species identification, and transcriptomics [18].

Up to now, the transcriptomic information of *A. cerana* has remained limited. Previous studies on the *A. cerana* transcriptome were based on second-generation high-throughput sequencing technology with short reads [5,6], which could not accurately identify alternative splicing or fusion genes. Moreover, previous studies focused only on transcripts from particular developmental stages, failing to provide a full picture of all transcripts from the entire life cycle of *A. cerana*. Therefore, in this study, we employed nanopore sequencing technology to conduct full-length transcriptome sequencing on samples from *A. cerana* queen, drone, and worker samples to obtain sequence information of all *A. cerana* mRNA molecules. Our present research yielded high-quality transcript sequences that can further refine the genomic sequence structure and annotation information associated with *A. cerana*.

## 2. Results

### 2.1. Transcriptome Sequencing Using Oxford Nanopore Technology

Transcriptome sequencing using the Oxford Nanopore platform was performed on queen, worker, and drone samples. After filtering out short fragments and low-quality reads, we obtained 48,440,954, 29,142,322, and 47,319,616 high-quality clean reads with average lengths of 1227, 1304, and 1185, respectively (Table 1, Figure S1). Subsequently, we removed rRNA sequences and obtained 46,283,004, 27,547,203, and 45,012,392 clean reads, which contained 38,813,295, 22,254,923, and 37,986,725 full-length transcripts, respectively. The full-length transcripts comprised 83.86%, 80.79%, and 84.39% of the total clean reads (excluding rRNA) (Table 1). Further filtering of redundant full-length transcripts yielded 100,927, 71,554, and 105,584 non-redundant full-length transcripts, with average lengths of 1521 bp, 1649 bp, and 1442 bp, respectively. By combining the Nanopore sequencing reads from the three samples, filtering out low-quality reads and rRNA sequences, and removing redundancy, we obtained a final set of 160,811 non-redundant full-length transcripts, with an average length of 1548 bp and a maximum read length of 10,874 bp (Table S1).

### 2.2. Structure Analysis and Functional Annotation of Novel Transcripts

The transcriptome sequencing in this study generated 160,811 non-redundant full-length transcripts, corresponding to 19,859 genes. Among these transcripts, 19,622 were from known genes, while 141,189 were novel, accounting for 12.20% and 87.80% of the total transcripts, respectively. A total of 130,367 transcripts were annotated using eight major databases (NR, COG, KOG, Pfam, Swiss-Prot, eggNOG, KEGG, GO), accounting for 81.07% of all transcripts (Table 2 and Table S2).

### 2.3. Unique Transcripts from Queen, Worker and Drone Data

**Figure 1:**
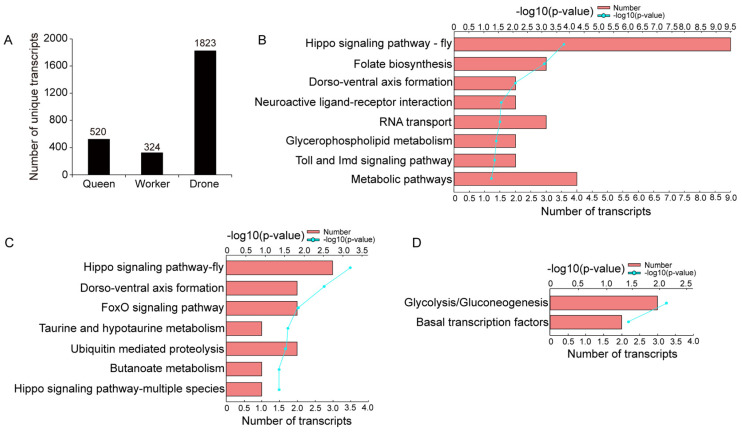
Unique transcripts isolated from queen, worker, and drone datasets. (**A**) Number of unique transcripts in queen, worker, and drone datasets. (**B**–**D**) show the significantly enriched KEGG pathways for the unique transcripts of the queen, worker, and drone bees, respectively.

A total of 520 transcripts were unique to the queen, 324 to the worker, and 1823 to the drone (Figure 1A, Table S3). KEGG analysis showed that these unique transcripts were significantly enriched in eight, seven, and two pathways, respectively (*p* < 0.05; Figure 1B–D). Of these, several pathways were related to honey bee caste differentiation, such as the “Hippo signaling pathway—fly”, “Toll and Imd signaling pathway”, “FoxO signaling pathway”, and “Hippo signaling pathway—multiple species”

### 2.4. AS Events

**Figure 2:**
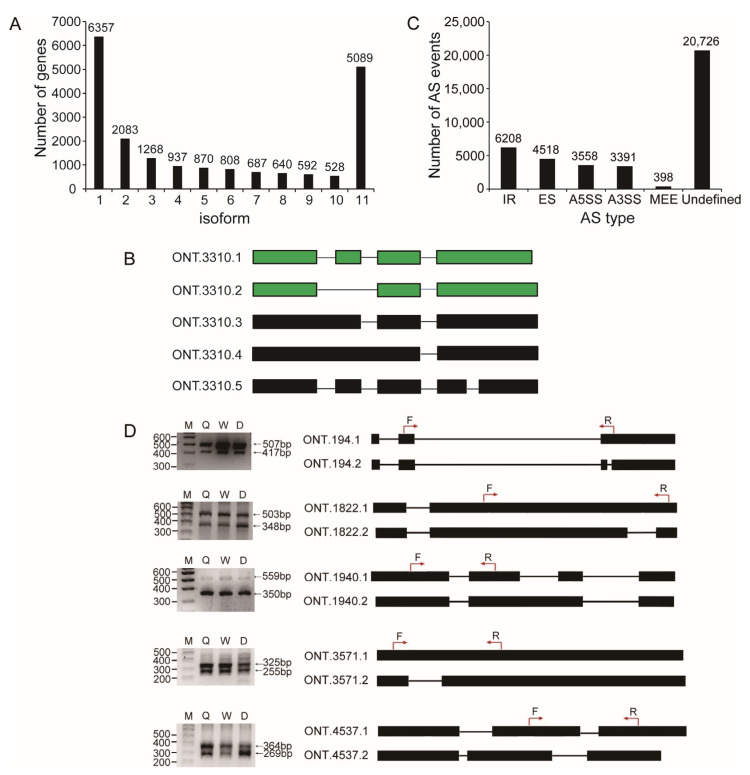
Alternative splicing events identified from the nanopore honey bee transcriptome. (**A**) Distribution of splice isoforms of genes. (**B**) Exon/intron structure of the five isoforms of the l(3)mbn gene. The isoform in green was from the NCBI reference transcript set. (**C**) The number of each type of alternative splicing event in this Nanopore dataset. (**D**) Verification of the alternative splicing events in five genes by RT-PCR. The exon/intron structure of each isoform of each gene is shown in the right panel. The filled boxes represent exons, and the lines represent introns. The locations of the PCR primers for each gene are indicated with red arrows. F: forward primer; R: reverse primer; M: marker; Q: queen; W: worker; D: drone.

Data analysis revealed that 6357 genes (32.01%) had one isoform, while 13,502 genes (67.99%) possessed two or more isoforms (Figure 2A). The *A. cerana* l(3)mbn (LOC107999844) gene contained five isoforms in our sequencing data, but only two isoforms were deposited in the GenBank database at NCBI (Figure 2B).

From the queen, worker, and drone datasets, 23,235, 17,024, and 30,507 AS events were identified, respectively (Figure S2). After combining the data from the three samples, a total of 38,799 AS events were detected from 5710 genes, including 6208 intron retention (IR), 4518 exon skipping (ES), 3558 alternative 5′ splice sites (A5SS), 3391 alternative 3′ splice sites (A3SS), 398 mutually exclusive exons (MEE), and 20,726 undefined AS events (Figure 2C). Among these, intron retention (IR) was the predominant type, consistent with findings in other animals and plants.

The accuracy of AS events for five genes was validated by RT-PCR. The results showed that the sizes of the PCR amplification fragments were consistent with the predicted isoforms (Figure 2D).

### 2.5. APA Sites

**Figure 3:**
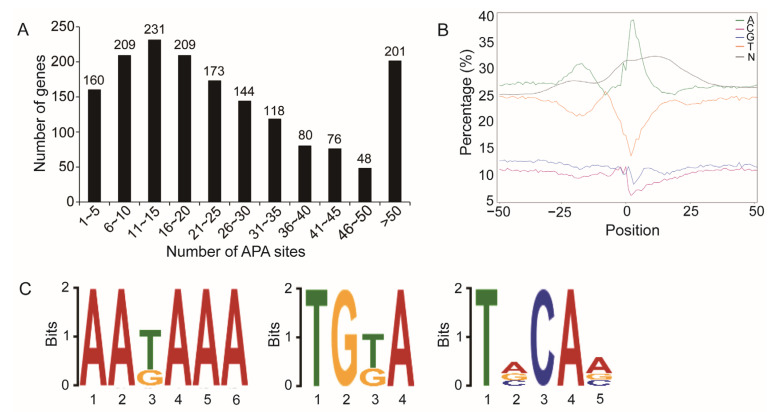
Alternative polyadenylation sites identified from the Nanopore honey bee transcriptome. (**A**) Distribution of the number of APA sites per gene. (**B**) Nucleotide composition of the regions 50 bp upstream and downstream of the poly(A) sites. (**C**) Conserved elements near the poly(A) sites predicted by DREME analysis.

Based on the analysis of 160,811 transcripts, a total of 44,243 APA sites at 1649 gene loci were identified, with an average of 26.83 poly(A) sites per gene. Most genes had more than one poly(A) site (Figure 3A). The nucleotide compositions 50 bp upstream and downstream of the poly(A) sites were enriched with adenine (A) and uracil (U) (Figure 3B). The top three conserved elements overrepresented in this region were AAKAAA, TGKA, and TVCAV (Figure 3C).

### 2.6. SSRs

**Figure 4:**
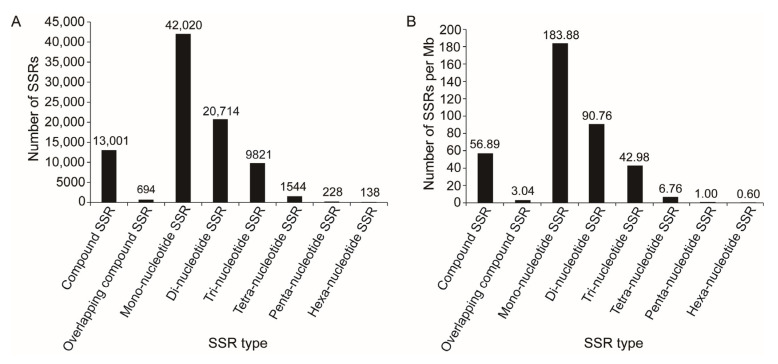
The number (**A**) and density (**B**) of each type of SSR identified from the transcripts.

A total of 88,187 SSRs from 52,625 transcripts were identified, including seven types of SSRs. Among them, mono-nucleotide SSR was the most common type and has the highest density on the transcripts (Figure 4).

### 2.7. LncRNAs

**Figure 5:**
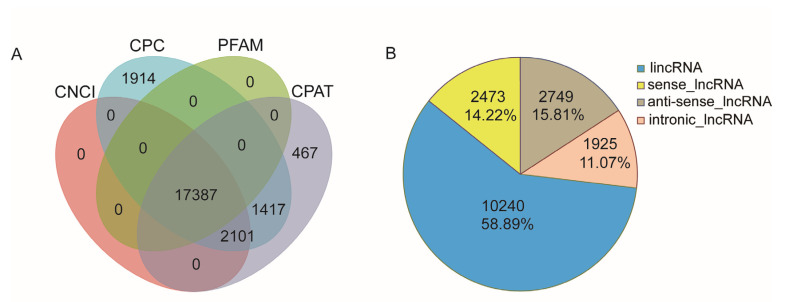
LncRNAs identified from the nanopore honey bee transcriptome. (**A**) Venn diagram showing the number of lncRNAs predicted by CPC, CPAT, PFAM, and CNCI. (**B**) Proportions of the four types of lncRNAs.

A total of 17,387 lncRNA transcripts, with a mean length of 999 nt, were predicted by CPC/CNCI/CPAT/Pfam analysis (Figure 5A). The noncoding transcripts identified using these four methods were intersected with the results of lncRNAs for subgroup analysis. Based on their positions on the *A. cerana* genome, all detected lncRNAs were subdivided into four types: 10,240 long intergenic noncoding RNAs (lincRNAs, 58.89%), 2749 antisense-lncRNAs (15.81%), 2473 sense lncRNAs (14.22%), and 1925 intronic lncRNAs (11.07%) (Figure 5B).

### 2.8. Differentially Expressed Transcripts (DETs) in the Reproductive Glands of A. cerana

We aligned the RNA-seq data from the queen ovaries, worker ovaries, and drone testes of *A. cerana* [6] to a non-redundant set of 160,811 transcripts and calculated the expression levels of each transcript in each sample. A total of 61,133 transcripts from 17,546 genes were found to be expressed in at least one of the samples. We utilized these genes and transcripts for differential expression analysis.

**Figure 6:**
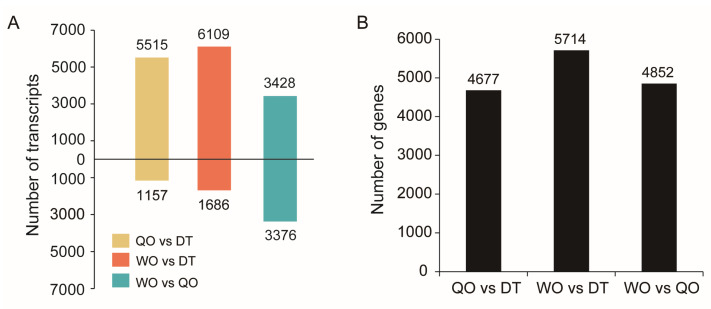
DETs (**A**) and their related genes (**B**) between queen ovaries (QO), worker ovaries (WO), and drone testes (DT).

In the comparison between queen ovaries and drone testes, we identified 6672 DETs related to 4677 genes (Figure 6A,B, and Table S4). Among these DETs, 5515 were upregulated in the queen ovaries, while 1157 were downregulated. These DETs were significantly enriched in 26 KEGG pathways (*p* < 0.05) (Table S5). In the comparison between worker ovaries and drone testes, we detected 7795 DETs related to 5714 genes (Figure 6A,B and Table S4). Of these DETs, 6109 were upregulated in the worker ovaries, and 1686 were downregulated. These DETs were significantly enriched in 19 KEGG pathways (*p* < 0.05) (Table S5). Comparing worker ovaries with queen ovaries, we identified 6804 DETs related to 4852 genes (Figure 6A,B and Table S4). Among these, 3428 DETs were upregulated in the worker ovaries, and 3376 DETs were downregulated. These DETs were significantly enriched in 12 KEGG pathways (*p* < 0.05) (Table S5).

## 3. Discussion

*A. cerana* is a widely cultivated bee species in Asia with significant economic value. In this study, we employed Nanopore sequencing technology to directly sequence the cDNA of *A. cerana*, resulting in the generation of full-length transcripts. The number of transcripts obtained was 3.31 times greater than the existing transcripts available in the NCBI database. Additionally, a substantial number of novel transcript isoforms were discovered. These transcripts greatly complement the existing genomic resources of coding genes and transcript sequences in *A. cerana*, providing crucial sequence information for further investigations into gene function in this species.

In this study, many transcripts unique to queen, worker, and drone bees were identified, which may be associated with the specific genetic characteristics of these three kinds of bees. For examples, two transcripts (ONT.1831.7 and ONT.1834.1) encoding cuticle proteins are found exclusively in queens, which may be associated with the development of queen cuticles, given the differences in body color and size between queens and the other castes (workers and drones); six transcripts (ONT.8878.1, ONT.8879.3, ONT.8879.4, ONT.8883.6, ONT.8883.7, and ONT.8883.9) encoding major royal jelly proteins are unique to workers, which is likely due to the fact that only workers have hypopharyngeal glands that express these proteins; three transcripts (ONT.13433.4, ONT.13433.5, and ONT.13709.1) encoding sperm flagellar proteins are unique to drones, which may be related to sperm motility.

Alternative splicing is an important mechanism for increasing protein diversity encoded by the genome in eukaryotic organisms. In human beings, more than 95% of genes can produce at least two isoforms through alternative splicing [10]. In this study, we found that approximately 67.99% of *A. cerana* genes exhibited alternative splicing, which is much higher than that observed in other insects, such as *Drosophila* [19], *Bombyx mori * [20], *Nilaparvata lugens* [21], and *Plutella xylostella* [22]. This finding suggests that alternative splicing is an important contributor for enhancing transcript diversity in *A. cerana*. Compared with our previous study in *A. mellifera* [23], the number of transcripts obtained from *A. cerana* in this study was greater than that of *A. mellifera* transcripts, but the number of AS events in *A. cerana* was much lower than in *A. mellifera*. This suggests that AS events are more frequent in the transcripts of *A. mellifera* than in those of *A. cerana*, and also suggests that there may be more types of AS events in a single gene of *A. mellifera* compared to *A. cerana*.

Alternative polyadenylation produces mRNA isoforms with different 3′ ends due to different terminal cleavage sites in nascent transcripts, thereby increasing transcriptome diversity. Moreover, APA can affect many aspects of mRNA post-transcriptional processing, including mRNA stability, localization, translation, and co-translational protein–protein interactions [24]. In *Drosophila* [25], worms [26], and zebrafish [27], approximately half of the genes contain more than two APA sites. In this study, we found a great amount of APA sites in *A. cerana*, and 98.12% of the genes related to APA in our transcriptome have two or more APA sites. It implies that APA is an important mechanism contributing to the diversity of the *A. cerana* transcriptome.

Furthermore, a total of 17,387 lncRNAs were identified in this study. LncRNAs are widely involved in various biological processes and are important components of the gene expression regulatory network in organisms [28]. It suggests that these lncRNAs may widely participate in various biological processes within Asian honey bees. Of these lncRNAs, most of them were classified as intergenic lncRNAs, which is consistent with findings in *Bombyx mori* [29], *Zeugodacus cucurbitae* [30], *Aedes albopictus* [31], and *Bactrocera dorsalis* [32]. This implies that intergenic regions of the honey bee genome have important regulatory functions in various biological processes in Asian honey bees.

Using all the transcripts obtained in this study as reference sequences, we identified many DETs between the queen ovary, worker ovary, and drone testis using RNA-seq data reported in a previous study [6]. The number of DET-related genes is more than 13 times the number of DEGs reported in that study [6], which suggests that many genes exhibit differential expression on certain splicing isoforms between these three tissues rather than at the entire gene level. For example, one of the transcripts of the *sxl* gene (a key gene in sex determination of *Drosophila melanogaster*), ONT.19423.21, showed expression difference between queen ovary and drone testis in our data, but the *sxl* gene did not display expression difference between them at the gene level. These findings provide important gene transcription information for unraveling the molecular mechanisms underlying the developmental differentiation of queen ovaries, worker ovaries, and drone testes.

Although we obtained a huge number of *A. cerana* transcripts using nanopore sequencing, we observed quite a number of unrecognized bases (denoted as N) in the transcript sequences. This is mainly caused by the relatively high sequencing error rate of nanopore sequencing technology. These unrecognized bases may hinder the accurate identification of certain alternative splicing sites, APA sites, and SSR sites, ultimately leading to an underestimation of their actual numbers. With the development and improvement of sequencing technology, due to the advantages of high throughput and low cost, we believe that nanopore sequencing technology will become an important sequencing method in the field of omics research.

## 4. Materials and Methods

### 4.1. Sample Source

The honey bees used in this experiment were *A. cerana cerana* raised in an apiary in the Honey Bee Research Institute at Jiangxi Agricultural University (28.46° N, 115.49° E). Sampling was conducted during the reproductive season of the bees in spring, from May to June. To capture the transcriptomic information of the three bee castes across different developmental stages and tissues, we collected samples from multiple time points and various organs/tissues of the queens, workers, and drones (Table S6). Initially, a healthy honey bee colony with a normal egg-laying queen was selected. The queen was confined to an empty comb for six hours to lay fertilized eggs in worker cells. Worker samples were collected at different developmental time points. After the workers emerged as adults, organs/tissues such as antennae, brain, hypopharyngeal glands, legs, muscle, midgut, fat body, and skin were collected. Queen samples were collected from the same colony, and the queen was confined to a comb to lay eggs for six hours. After the hatching of the eggs, the 1-day-old larvae were transferred to queen cells for queen rearing. Samples of queens at different developmental time points and various organs/tissues of newly emerged queens were collected. The sampling method for drones was the same as that for workers. A virgin queen that had not mated was used to establish a colony. After the queen started laying eggs normally, she was confined to an empty comb in a drone comb to lay unfertilized eggs for six hours. Samples of drones at different developmental time points, as well as various organs/tissues of newly emerged drones, were collected. All samples were rapidly frozen and stored in liquid nitrogen for further use.

### 4.2. Library Construction and Sequencing

Total RNA was extracted from each of the above-collected *A. cerana* samples using TRIzol Reagent (CWBIO Biotechnology Co., Ltd., Taizhou, China). The concentration and integrity of RNA were measured using Nanodrop2000 (Thermo Fisher Scientific, Wilmington, DE, USA) and the Agilent Bioanalyzer 2100 system (Agilent Technologies, Santa Clara, CA, USA). Then, RNA samples from the same caste were pooled in equal quantities, and 1 µL of the mixed RNA was subjected to library construction using the cDNA-PCR Sequencing Kit provided by Oxford Nanopore Technologies Company (Oxford, UK) according to its protocol. The final cDNA libraries were loaded onto FLO-PRO002 flow cells and run on the PromethION48 platform at Biomarker Technology Company (Beijing, China).

### 4.3. Analysis of the Raw Data

The raw sequencing data were subjected to base calling using the Guppy v6.0 software in the MinKNOW v2.2 package [33]. Subsequently, the fast5 format data were converted to fastq format. Low-quality sequences with a length of less than 200 bp and a Qscore of less than 6 were filtered out. Next, rRNA sequences were removed by aligning against the rRNA database, and full-length sequences were identified based on the presence of primers at both ends (Figure S3). The full-length sequences were aligned to the *A. cerana* reference genome ApisCC1.0 (GenBank accession number: GCA_002290385.1) using Minimap2 v2.16 software [34]. Clustering was performed based on the alignment information, and consistent sequences were obtained using the Pinfish pipeline. These consistent sequences were again aligned to the reference genome using Minimap2 v2.16 software to remove redundant sequences. The redundant sequences were filtered out based on an identity threshold of less than 0.9 and coverage of less than 0.85, and alignments with only the differential 5′ exon were merged to obtain the final non-redundant transcript sequences.

### 4.4. Analysis of Alternative Splicing

The Astalavista v3.2 software [35] was used to identify five types of AS events present in each sample, including exon skipping (ES), intron retention (IR), alternative donor site (AD), alternative acceptor site (AA), and mutually exclusive exon (MEE). The numbers of each type of AS event in the transcripts were then counted.

### 4.5. Identification and Analysis of Novel Transcripts

Novel genes and transcripts were identified by comparing the full-length transcripts with the known transcripts in the *A. cerana* genome using gffcompare v0.9.8 software [36]. Subsequently, TransDecoder v3.0.0 software [36] was employed to predict the coding regions and corresponding amino acid sequences of the newly discovered transcripts. To obtain comprehensive annotation information for the novel transcripts, sequence alignments were performed against the NR, SwissProt, GO, COG, KOG, Pfam, eggNOG, and KEGG databases. This comprehensive comparative analysis provided valuable annotations for the transcripts, encompassing functional annotations, protein domains, and associations with biological pathways. TransDecoder v3.0.0 software [37] was used to predict coding sequences (CDS) for the novel transcripts, resulting in the identification of both amino acid and nucleotide sequences for the coding regions.

### 4.6. Identification of APA Sites and SSRs

The TAPIS pipeline [38] was utilized for the identification of APA sites in precursor mRNAs. The upstream and downstream 50 bp sequences of polyadenylation sites for all transcripts were analyzed using DREME v4.11.3 [39] to identify motifs within this region. Additionally, MISA v1.0 software [40] was employed for the analysis of SSRs. Seven types of SSRs were identified: mono-nucleotide, di-nucleotide, tri-nucleotide, tetra-nucleotide, penta-nucleotide, hexa-nucleotide, and compound SSR.

### 4.7. Prediction of LncRNAs

The newly discovered transcripts were subjected to lncRNA prediction using four different methods: CPC (Coding Potential Calculator) [41], CNCI (Coding-Noncoding Index) [42], CPAT (Coding Potential Assessment Tool) [43], and Pfam [44]. The noncoding transcripts identified by these four analysis tools were intersected to obtain a set of transcripts for subsequent lncRNA analysis. Based on the position of lncRNAs in the annotation information (gff) of the reference genome, the lncRNAs were then classified into different categories.

### 4.8. Identification of Differentially Expressed Transcripts

Transcriptome sequencing data for the ovaries of *A. cerana* queens, ovaries of workers, and testes of drones were downloaded from the Sequence Read Archive (SRA) database at the National Center for Biotechnology Information (NCBI) under accession numbers SRX9791501, SRX9791502, and SRX9791511-SRX9791514. The complete transcriptome sequences obtained in this study were used as reference sequences. Differential expression analysis was performed to identify transcripts with significant expression differences. The criteria for selecting differentially expressed transcripts were set as |log2(fold change)| ≥ 1 and *p* < 0.05. GO and KEGG enrichment analyses were conducted to explore the functional annotations and pathways associated with the differentially expressed transcripts.

### 4.9. RT-PCR

Five genes were randomly selected to verify the existence of the obtained transcripts using RT-PCR. Newly emerged queen, worker, and drone bees were sampled, respectively, for total RNA extraction and cDNA syntheses. Primers were designed using Primer Premier 5.0 (PREMIER Biosoft International, San Francisco, CA, USA) to amplify all the detected transcripts of each gene based on full-length sequences (Table S7). The PCR amplification reaction solution consisted of 10 µL 2× Taq PCR StarMix (Dye), 3 µL cDNA, 1 µL forward primer, 1 µL reverse primer, and 5 µL sterile water. PCR conditions were as follows: pre-denaturation at 94 °C for 3 min; 35 amplification cycles of denaturation at 94 °C for 30 s, 60.0 °C for 30 s, 72 °C for 45 s, and final elongation at 72 °C for 10 min. The PCR amplification was performed using a T100 Thermal Cycler (BIO-RAD, Hercules, CA, USA). PCR products were verified by electrophoresis on a 1.2% agarose gel and then confirmed by Sanger sequencing.

## 5. Conclusions

Through nanopore single-molecule sequencing, we obtained 160,811 full-length transcript sequences in *A. cerana*, and most of them were novel transcripts. Based on these transcripts, a large number of AS events, APA sites, SSRs, and lncRNAs were identified. Our study greatly enriched the transcriptome data of *A. cerana*, providing valuable insights into its transcriptomic landscape. Furthermore, our findings contribute to a better understanding of the genetic mechanisms underlying the diverse biological processes in this important bee species.

## Figures and Tables

**Table 1:** The summary of sequencing results.

Sample	Clean Reads	Base	Mean Length	Clean Reads (Except rRNA)	Flnc Reads	Flnc Ratio	Non-Redundant Flnc Reads
Queen	48,440,954	59,474,666,129	1227	46,283,004	38,813,295	83.86%	100,927
Worker	29,142,322	38,028,781,394	1304	27,547,203	22,254,923	80.79%	71,554
Drone	47,319,616	56,083,323,832	1185	45,012,392	37,986,725	84.39%	105,584

**Table 2:** Summary of the annotation results of all the transcripts.

Database	Transcript
Total	160,811 (100.00%)
NR	130,198 (80.96%)
COG	43,349 (26.96%)
KOG	92,473 (57.50%)
Pfam	4526 (2.81%)
Swiss-Prot	84,235 (52.38%)
eggNOG	120,122 (74.70%)
GO	90,872 (56.51%)
KEGG	82,740 (51.45%)
Unannotated	30,444 (18.93%)

## Data Availability

The clean reads obtained by nanopore single-molecule sequencing have been submitted to the Sequence Read Archive (SRA) database and are available from NCBI under BioProject number PRJNA1116335.

## Supplementary Materials

The following supporting information can be downloaded at: https://www.mdpi.com/article/10.3390/ijms251910833/s1.
