# Supplementary material for: Full-Length Transcriptome Profile of Apis cerana Revealed by Nanopore Sequencing
Source: Int J Mol Sci. 2024 Oct 9;25(19):10833. doi: 10.3390/ijms251910833 (PMC11476444; doi:10.3390/ijms251910833)
Supplement: Supplementary file 1 [file ijms-25-10833-s001.zip › TableS6.pdf]

Table S6. The samples from different developmental time points and organs/tissues of *A.cerana*.

Sample	Developmental time points and organs/tissues
Queen	0h egg, 24h egg, 48h egg, 72h egg, 2d larvae, 4d larvae, 5d larvae, 3d pupae, 5d pupae, 7d pupae, newly emerged queen, sexually mature queen; and antenna, brain, hypopharyngeal gland, muscle, leg, middle intestine, skin and ovary of newly emerged queen.
Worker	0h egg, 24h egg, 48h egg, 72h egg, 2d larvae, 4d larvae, 6d larvae, 3d pupae, 6d pupae, 8d pupae, new emerged worker bee, nurse bee, forger bee; and antenna, brain, hypopharyngeal gland, muscle, leg, middle intestine and skin of newly emerged worker
Drone	0h egg, 24h egg, 48h egg, 72h egg, 2d larvae, 5d larvae, 3d pupae, 8d pupae, 12d pupae, new emerged drone, sexually mature drone; and antenna, brain, hypopharyngeal gland, muscle, leg, middle intestine, skin and testis of newly emerged drone
