# Supplementary material for: Full-Length Transcriptome Profile of Apis cerana Revealed by Nanopore Sequencing
Source: Int J Mol Sci. 2024 Oct 9;25(19):10833. doi: 10.3390/ijms251910833 (PMC11476444; doi:10.3390/ijms251910833)
Supplement: Supplementary file 1 [file ijms-25-10833-s001.zip › TableS7.pdf]

Table S7. The primers for verification of alternative splicing events

Gene	Primer sequences
ONT.194	F: TTGAAGCAGTCCACGCTAGG<br>R: TTACGCGACCCACGAAAGAA
ONT.1822	F: GGGCAGCAACAACAATTGGT<br>R: TTGAGGGGGTTGTACGGTTG
ONT.1940	F: TCCAGGGCAGCAACAAGTAG<br>R: CCTCTTCTCCCGGTTGCTTT
ONT.3571	F: ATGGGGTAGGCGAGGATGTT<br>R: TTCTCGCCGTGATGCTATTC
ONT.4537	F: CGCCATTCTCCAACAACAGTC<br>R: AAGCCCCTTCCTCGTATTCA
