# Supplementary figures and images for: Full-Length Transcriptome Profile of Apis cerana Revealed by Nanopore Sequencing
Source: Int J Mol Sci. 2024 Oct 9;25(19):10833. doi: 10.3390/ijms251910833 (PMC11476444; doi:10.3390/ijms251910833)

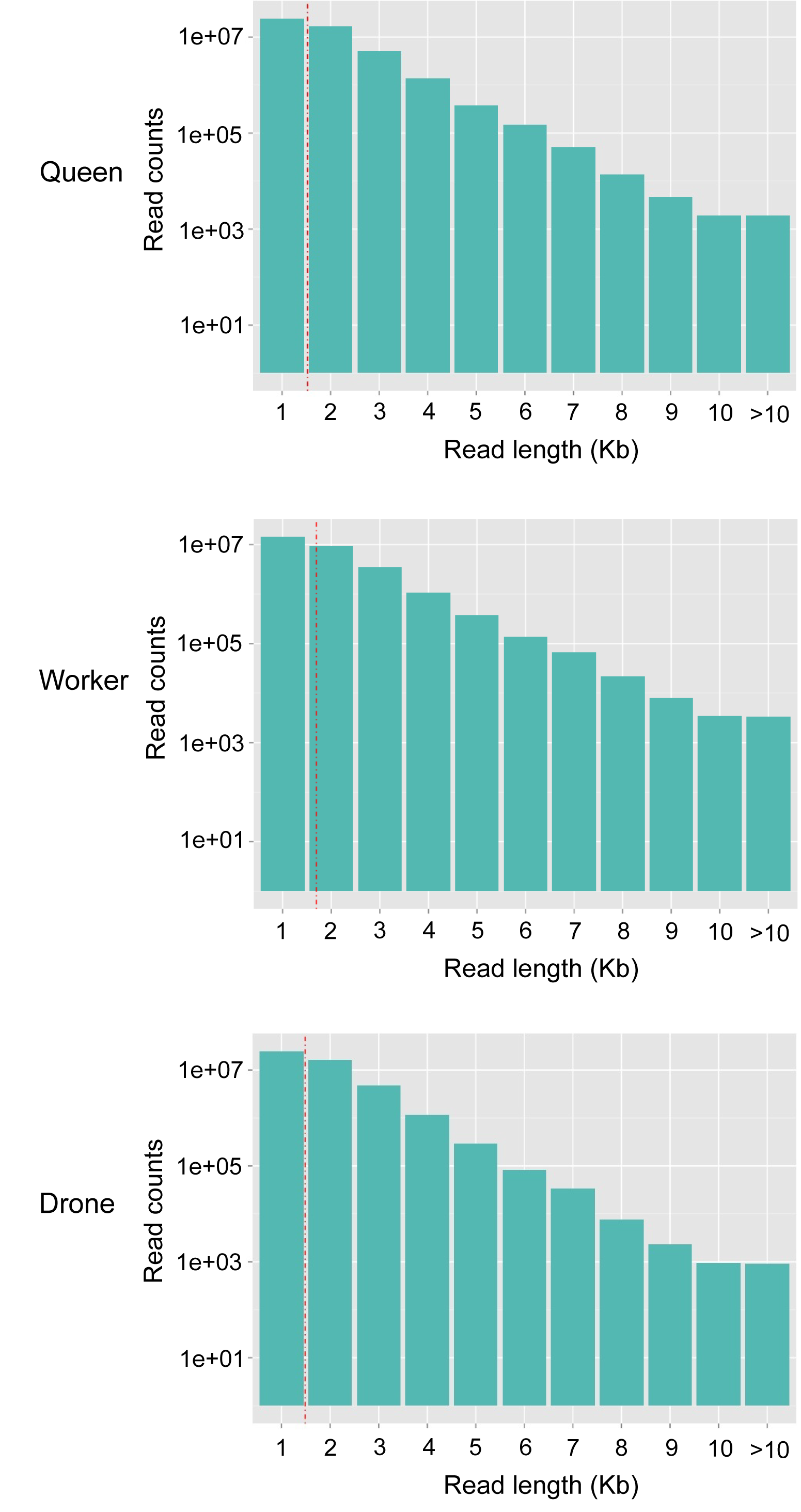

Supplement: Supplementary file 1 [file ijms-25-10833-s001.zip › FigureS1.tif]

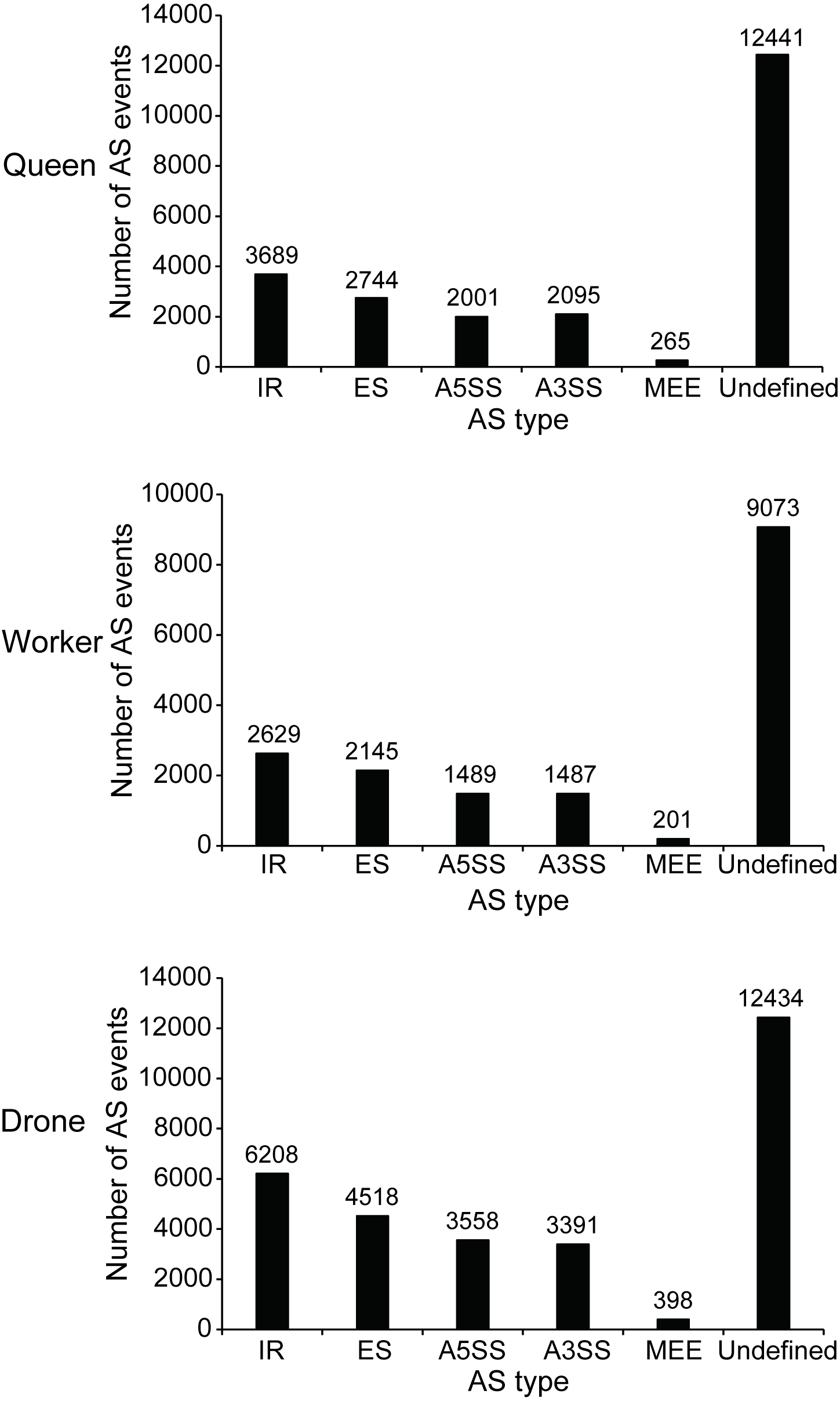

Supplement: Supplementary file 1 [file ijms-25-10833-s001.zip › FigureS2.tif]

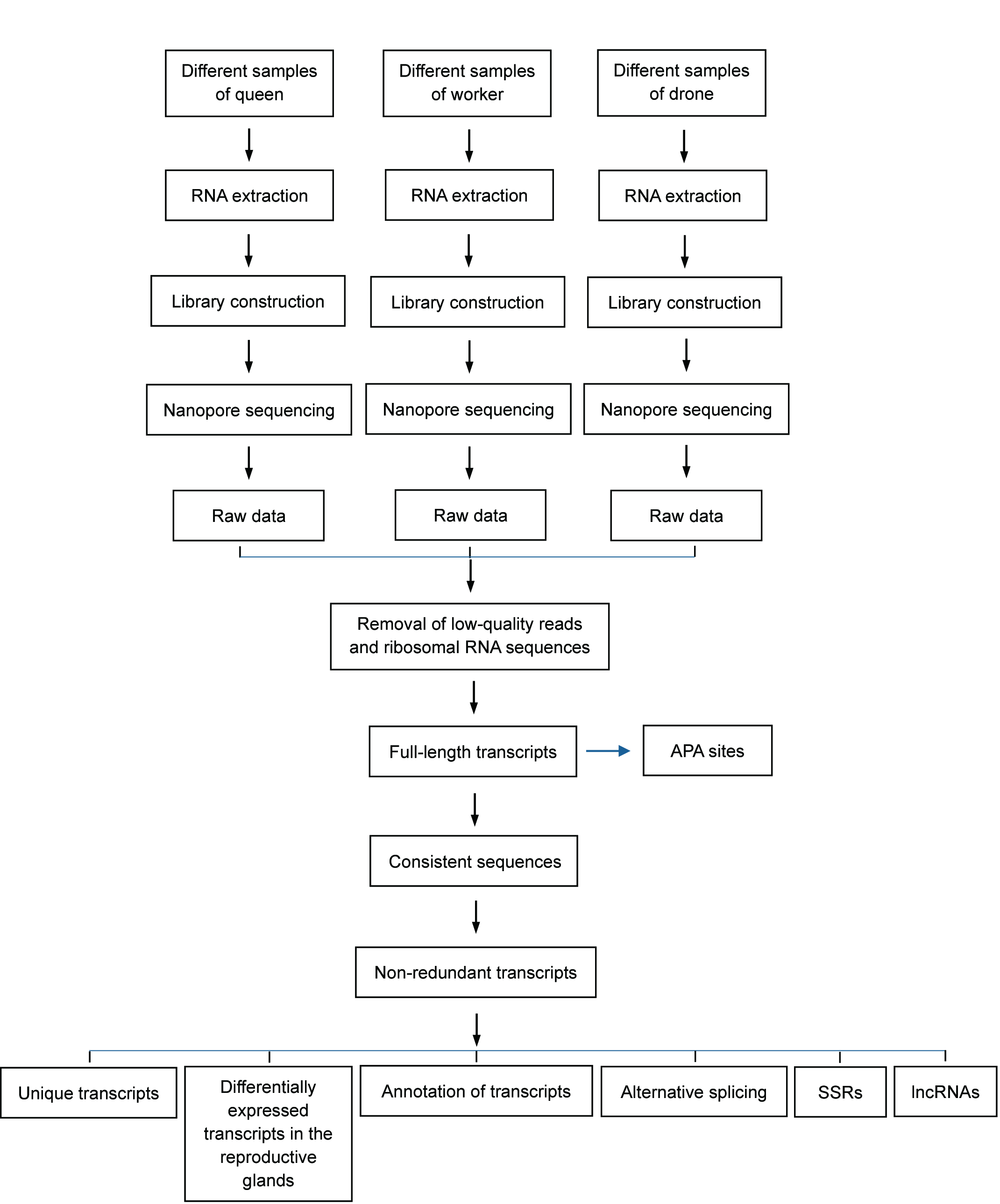

Supplement: Supplementary file 1 [file ijms-25-10833-s001.zip › FigureS3.tif]
